# Lower limbs micro-loading acutely attenuates repeated change-of-direction performance in male youth during small-sided soccer games

**DOI:** 10.1186/s13102-023-00778-1

**Published:** 2023-12-18

**Authors:** Mohamed Amine Ltifi, Hassane Zouhal, Ismail Laher, Ayoub Saeidi, Karuppasamy Govindasamy, Urs Granacher, Ridha Aouadi, Abderraouf Ben Abderrahman

**Affiliations:** 1 Higher Institute of Sport and Physical Education of Ksar-Said, University of Manouba, Tunis, Tunisia; 2 Laboratoire Mouvement Sport, Santé - EA 1274, Univ Rennes, M2S, Rennes, F-35000 France; 3 Institut International des Sciences du Sport (2IS), Irodouer, 35850 France; 4 Department of Anesthesiology, Pharmacology and Therapeutics, The University of British Columbia, Vancouver, Canada; 5 Department of Physical Education and Sport Sciences, Faculty of Humanities and Social Sciences, University of Kurdistan, Sanandaj, Kurdistan Iran; 6 Department of Physical Education and Sports Sciences, College of Science and Humanities, SRM Institute of Science and Technology, Kattankulathur, 603 203 Tamil Nadu India; 7 Department of Sport and Sport Science, Exercise and Human Movement Science, University of Freiburg, Freiburg, Germany; 8 Tunisian Research Laboratory “Sports Performance Optimization”, National Center of Medicine and Science in Sports (CNMSS), Tunis, Tunisia; 9 Research Laboratory (LR23JS01) “Sport Performance, Health & Society” Higher Institute of Sport and Physical Education of Ksar Said, University of Manouba, Tunis, Tunisia

**Keywords:** Additional weight, Change-of-direction speed, Football match play, Wearable resistance

## Abstract

**Background:**

Soccer players often wear light-weighted wearable resistance (WR) attached to different body parts during the warm-up period with the aim to improve measures of physical fitness. However, the effect of WR on physical performance is unknown. This study evaluated the effects of WR with different micro-loadings on repeated change-of-direction (RCoD) performance while executing small-sided soccer games (SSG).

**Methods:**

Twenty male soccer players aged 16.0 ± 1.5 years (body mass 74.0 ± 7.4 kg, body-height 175.0 ± 10.0 cm) volunteered to participate in this study. Following a within-subject study design, players performed four specific warm-up protocols in randomized order with a rest of 72 h between protocols: (1) WR micro-loadings with 0.1% of body mass (WR0.1); (2) WR micro-loadings with 0.2% of body mass (WR0.2); (3) WR micro-loadings with 0.3% of body mass (WR0.3); (4) no WR (control = CONT). After the warm-up protocols, players performed 2 sets of 20-min SSG. The RCoD was collected at the 8th min of SSG (SSG 1–8 min), the 15th min of SSG1 (SSG1-15 min), and at the 15th min of SSG2 (SSG2-15 min). Outcomes included mean and total RCoD indices (i.e., mean time and total time for each condition).

**Results:**

Based on the outcomes of a two-way repeated measures analysis of variance (ANOVA), WR0.1 and WR0.2 were more effective than control in dampening the decrease of RCoD’s total time during SSG1-8 min, and SSG2-15 min (small ES: 0.24–0.35; *p* < 0.05). However, no significant differences were observed between WR0.3 and control. In addition, WR0.1 and WR0.2 significantly affected the decreases in RCoD’s mean best time during SSG1 and SSG2 which was observed in the unloaded condition (CONT) and consequently displayed a lower rate of RCoD performance decrease.

**Conclusion:**

This study reports that wearing lower extremity WRs with micro-loads of 0.1% or 0.2% of body mass attenuates physical fatigue indicated in attenuated RCoD performance while executing SSG.

## Introduction

Soccer is a multidirectional team-sport [1, 2] where complex sport-specific skills and cognitive tasks occur over a minimum of 90 min [3]. Playing soccer involves constant changes in the intensity of a variety of activities, including frequent standing, walking, running, and sprinting with jumping, as well as numerous high-speed displacements with repeated turns, twists, and explosive change-of-directions (CoD) [2]. Consequently, repeated CoD is an important feature of assessment [4] and a key factor determining sporting success in adult as well as young soccer players [5].

Small-sided soccer games (SSGs) are widely recognized as efficient training methods for competitive games [6–8]. The SSGs achieve desired training results [9] by developing technical and tactical aspects of the game, while also improving a number of physical capacities such as specific endurance, strength/power qualities, and agility in a match-related context [10]. Playing a soccer match entails ~ 600 turns from 0 to 90 degrees and ~ 95 turns of angles larger than 90 degrees [11, 12], all of which are also present in SSGs.

Warm-ups are designed to prepare the body for specific sport movements and enhance neuromuscular qualities of athletic performance without inducing fatigue [13]. Dynamic soccer warm-ups are a critical component of training and competition [14]. Different loaded conditioning strategies are also used to acutely improve soccer players’ related explosive performances [15, 16]. Proposals have been suggested to promote safe and pragmatic conditioning tools such as incorporating wearable resistance (WR) in standard soccer warm-up routines [15]. WR is routinely used in track and field training, particularly in sprint training by adding a micro-load during sport specific activities [17].

Using WR consists of attaching an external load to different segments of the body such as the lower limbs [18], ankles [19], and arms [20] while avoiding adverse effects on motor skill performance [21].

Given the practical and the ecological features of the WR, it could be easily used in many facets of soccer training programs. For instance, a study by Bustos et al. [15] reported that calf-loaded WR (200-to 600-g load) improved maximal horizontal (e.g., 10- and 20-m sprints) but not vertical (e.g., countermovement jump) performance during warm-ups in elite adolescent soccer players. Another study by Feser et al. [22] suggested that shank WR amplifies the degree of distinction of a soccer training protocol itself and that coaches could increase in-session workloads during periods of low training volume. Nevertheless, and despite the recent gain in popularity of WR, little is known about the potential efficacy of lower limbs WR warm-up as well as the appropriate magnitude and the choice of load placement [17].

The efficacy of using WR as a conditioning activity to improve soccer-related performances during the match is yet to be explored. The challenge is to load the limbs in such a way that the beneficial effects of the conditioning activity remain throughout the game. Therefore, our objective was to examine the extent to which different pre-conditioning lower limbs WR micro-loading protocols influence fluctuations in athletes’ repeated CoD performance while performing small-sided games in young soccer players. Based on previous findings that lower limbs WR loading (1 − 5% of body mass) with different loads had an acute effect upon CoD performance in male soccer players [23], we hypothesized that light WR (0.1, 0.2 and 0.3% of body mass) applied at the lower limbs improve repeated CoD performance during SSG.

## Methods

### Participants

Twenty post-puberal soccer players aged 16.0 ± 1.5 years (body mass 74.0 ± 7.4 kg, body-height 175.0 ± 10.0 cm) voluntarily participated in this study. Pubertal development was verified by a trained pediatrician using the Tanner stages and age at peak-height-velocity (PHV) [24–26]. According to the pediatrician’s classification and age at PHV, participants were rated Tanner stage 5 and were 2.0 years post PHV, which is indicative of post-puberty. Somatic maturity was assessed using age at peak-height-velocity (PHV) and the Mirwald et al. (2002) approach. The following regression equation was applied for male youth: (PHV = -7.999994 + [0.0036124 × age × height]). This equation has previously been validated by Mirwald et al. [26].

All participants had a background of at least ten years of systematic soccer training involving four training sessions, including one weekly workout in the gym and a soccer match on the weekend throughout the soccer season. Participants in the study had no prior experience with WR. Based on the players’ baseline characteristics including anthropometric data and the history of previous injuries which were given by the medical staff (team physician and physiotherapist) for each individual player. None of the players suffered from any musculoskeletal disorders during the three months prior to the study. Verbal and written informed consent from participants and their legal representatives were obtained after an explanation of the experimental protocol and its potential benefits and harms. All procedures were approved by the local Institutional Review Committee of the Higher Institute of Sport and Physical Education, Ksar Said, Tunisia (UR17JS01). The protocol was carried out in accordance with the latest version of the Declaration of Helsinki.

### Procedures

**Fig. 1:**
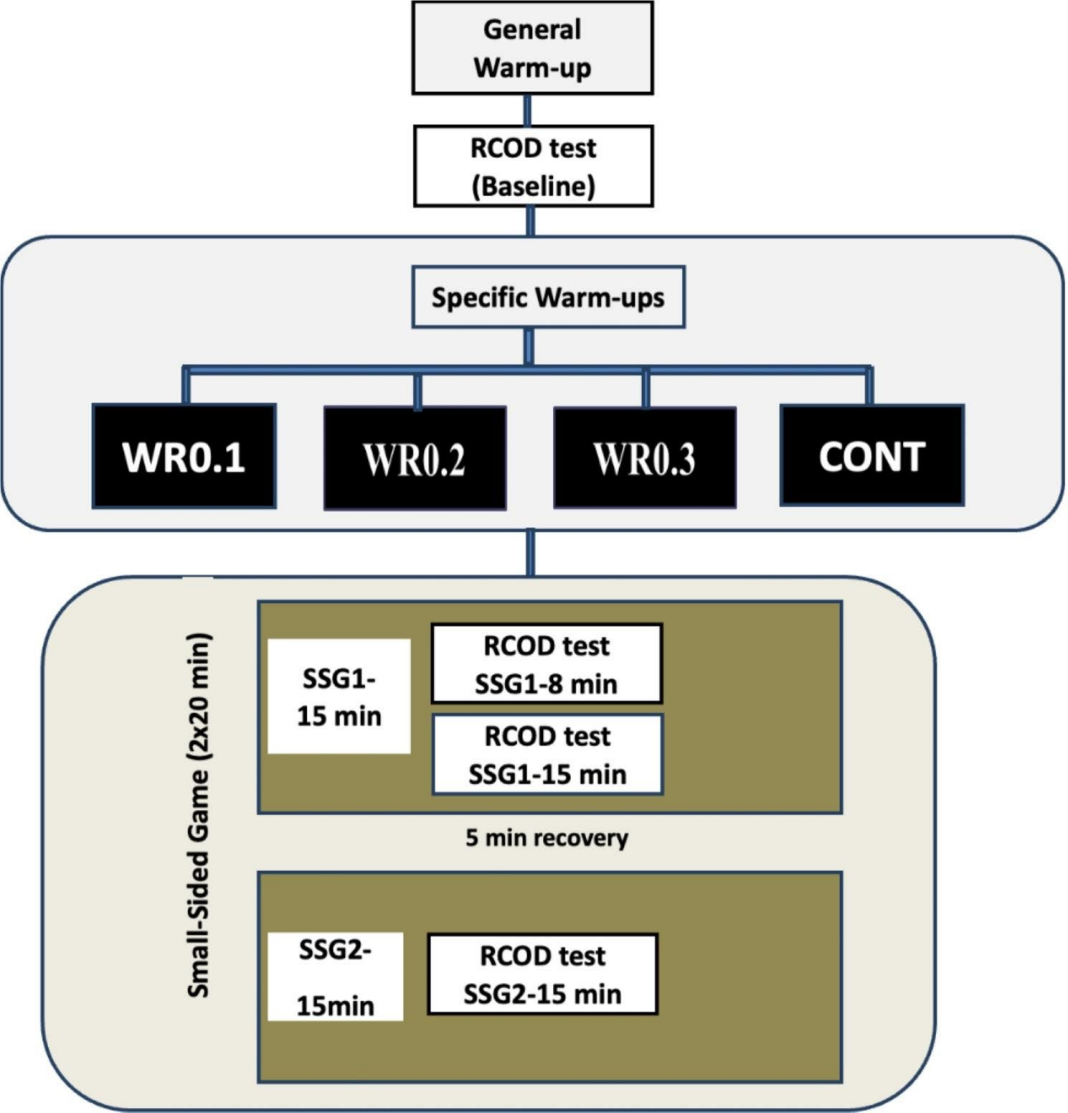
Schematic representation of the experimental design. Repeated CoD indicates repeated change of direction; SSG1: first half of Small-Sided Game; SSG2: Second Half of Small-Sided Game

Participants undertook three familiarisation and four test sessions. The first orientation session involved collecting anthropometric data (age, body mass, stature, and percentage of body fat), while participants were familiarized with the full experimental warm-up procedures and with the repeated CoD testing protocol during the second and the third orientation sessions. One week after the completion of the third preparatory session, participants performed four testing sessions in randomized order across two consecutive weeks (two tests per week) with 72 h rest in-between tests. Each test session began with a standardized warm-up consisting of a 5-minute general warm-up followed by 5 min of dynamic stretching [27]. Participants were then asked to perform one baseline repeated CoD trial (Fig. 1) before undertaking four experimental warm-up conditions in randomized order.

The experimental conditions consisted of the participants undertaking 10 min of a specific warm-up, including WRs attached to the ankle with magnitudes corresponding to either 0.1% (WR0.1), 0.2% (WR0.2), and 0.3% (WR0.3) of the athlete’s body mass (BM), and an unloaded control condition (CONT). A post-intervention repeated CoD performance was collected during the first (SSG1), and the second (SSG2) halves of the 2 × 20 min SSG. For the first test session, the players were randomly assigned to perform one of four experimental warm-up conditions using an online random number generator (Research Q6 Randomizer [version 4.0]). The remaining test sessions were performed in a cyclic order: [1] unloaded [2], WR0.1; [3] WR 0.2; [4] WR0.3 of BM and so on. For WR0.1, WR0.2, and WR0.3, the WRs were only worn during the later phase of the specific warm-up (exercises 9–11) (Table 1) [28]. The three conditioning loaded exercises consisted of: [1] exercise 9: running in a straight line over a distance of 10 m, repeated twice, with 10-s recovery (90 s); [2] exercise10: running in a straight line over a distance of 15 m, repeated 3 times, with 10-s recovery (2 min); and 3) exercise 11: running in a zigzag form over a distance of 20 m, repeated twice, with 10-s recovery (2 min). The CONT condition consisted of the subjects performing exercises 9-10-, and 11 without wearing WRs (unloaded).

**Table 1:** Complete warm-up protocol performed by participants [28]

**Phase 1: General warm-up**	
Exercise 1:	Light stroke (3 min).
Exercise 2:	Lateral movements (1 min).
Exercise 3:	Back stroke (1 min)
**Phase 2: Dynamic stretching**	
Exercise 4:	Walking forward with knee elevations to the chest and right trunk (1 min).
Exercise 5:	Walking with leg beats forward, the knee is held in full extension, and the foot is held in neutral position (1 min).
Exercise 6:	Walking forward with lateral elevation of the knee, the leg is bent at 90° “hedge position” (1 min).
Exercise 7:	Walking forward with heel to the buttocks (1 min).
Exercise 8:	Walking forward with flowing feet. Raising the body as high as possible on the tip of the foot keeping the trunk straight (1 min).
**Phase 3: Specific warm-up** ^**a**^	
Exercise 9:	Running in a straight line over a distance of 10 m, repeated twice, with 10-s recovery (90 s).
Exercise 10:	Running in a straight line over a distance of 15 m, repeated 3 times, with 10-s recovery (2 min).
Exercise 11:	Running in a zigzag form over a distance of 20 m, repeated twice, with 10-s recovery (2 min).

Note: ^**a**^ For WR0.1, WR0.2, and WR0.3, the WRs were only worn during the later phase of the specific warm-up (exercises 9–11)

The SSG consisted of two halves of 20 min (SSG1, and SSG2) separated by a 5-minute passive rest period. The repeated CoD was collected at 3 time points during the two halves of the soccer game: during SSG1 ((at the 8th min [SSG1-8 min]), and the 15th min [SSG1-15 min]), and during SSG2 (at the 15th min [SSG2-15 min]) (Fig. 1) so that each participant completed a single repeated CoD trial at each testing assessed time point during SSG1 and SSG2. The SSG were chosen as a replacement of a soccer match as they reproduce and improve soccer-related key variables [29, 30], and performances [31–33] in a time-efficient manner [9].

The repeated CoD test was chosen according to the validated protocol of Wong et al. [34], consisting of 6 maximum repetitions of 20-m sprints (four 100° CoD, every 4 m) interspersed by 25 s of active recovery. Best time (in seconds) and total time (in seconds) were used as performance indices of repeated CoD test. The repeated CoD performance was chosen as it appears to induce physiological responses similar to intense periods of soccer matches [35]. Players with better average repetition times usually tended to be those who play for longer periods [35]. Participants utilized a professional wearable lower limbs resistance (Adjustable Loading Weighted Leg Strap for Exercise Training) attached to the ankle. Investigations took place during the spring season (March-April 2022) between 4.00 and 6.00 p.m. Testing was grouped and conducted in a cyclical manner to complete trials as close together within a 2 h time span to avoid possible between-trial circadian variations in repeated CoD expression.

### Statistical analysis

Differences between experimental conditions were assessed using two-way repeated measures analyses of variance (ANOVA) and Bonferroni-corrected paired *t*-tests to analyze the effects of four experimental conditions and three time points on repeated CoD performance (i.e., total time and peak time). Data were analyzed using SPSS, V 17.0, software for windows (SPSS, Inc., USA, IBM Corp) and expressed as means and standard errors. The normality of the distributions was determined using the Kolmogorov-Smirnov test, and sphericity homogeneity was verified using Mauchly’s test and the Levene’s test, respectively. Greenhouse-Geisser corrections were used when the assumption of sphericity was violated. Statistical significance for all the tests was accepted at p<0.05. Bonferroni-adjusted pairwise post-hoc comparisons were used if significant interactions were detected in ANOVA calculations.

Baseline repeated CoDs were consistent across sessions (intra-class correlation coefficient [ICC] = 0.90). The absolute reliability, measured by the coefficient of variation (CV%), was calculated by dividing the SEM and the sum of the average attempts and multiplied by 100 [36]. Estimates of power and effectsize indices (Cohen’s d) were calculated to assess the magnitude of the effects [37]. Effect sizes (ES) were described as trivial (≤ 0.2), small (0.2 ≤ 0.5), medium (0.50 ≤ 0.8) and large (> 0.8). ES were calculated by determining the mean difference between conditions, and then dividing the result by the pooled standard deviation [37]. Inter-trial and intersession reliability of repeated CoD were determined and mean differences (with 95% confidence intervals [Cis]) were computed.

## Results

Pairwise analysis of repeated CoD performance total and mean times revealed no significant differences between experimental conditions and test sessions (*p* > 0.05). Total and mean repeated CoD time indices displayed strong inter-trial (ICC = 0.90–0.93; CV%: 1.64–3.21) and intersession reliability (ICC = 0.88–0.92; CV%: 3.47–4.08). Repeated CoD performance of both loaded (WR0.1, and WR0.2) and unloaded (CONT) conditions during pre- (baseline), and post- (SSG1-8 min, SSG1-15 min, and SSG2-15 min) warm-up interventions are presented in Table 2.

**Table 2:** Repeated CoD performances of WR0.1, WR0.2, WR0.3, using unloaded (CONT) and baseline conditions

Baseline	CONT	WR0.1	WR0.2	WR0.3
27.59 ± 1.05	28.81 ± 1.52^a^	28.60 ± 1.15^**b**^	28.71 ± 1.24^**b**^	28.89 ± 1.24

**Note**: ^a^significantly different from Baseline (*P* < 0.05), ^b^significantly different from CONT (*P* < 0.01)

ANOVA showed a significant main effect of time in both loaded and unloaded conditions for both mean and total times. Succeeding, post-hoc tests revealed significant decreases (*p* < 0.05) in post-warm-up intervention repeated CoD performance (i.e., for both mean and total times) (Table 2). However, when compared to CONT condition, a significant effect (*p* < 0.05) was observed with WR0.1 and WR0.2 being more effective in dampening the decrease of repeated CoD performance (increase of repeated CoD times) during SSG1-8 min, and SSG2-15 min (small ES: 0.24–0.35; (*p* < 0.05).

**Fig. 2:**
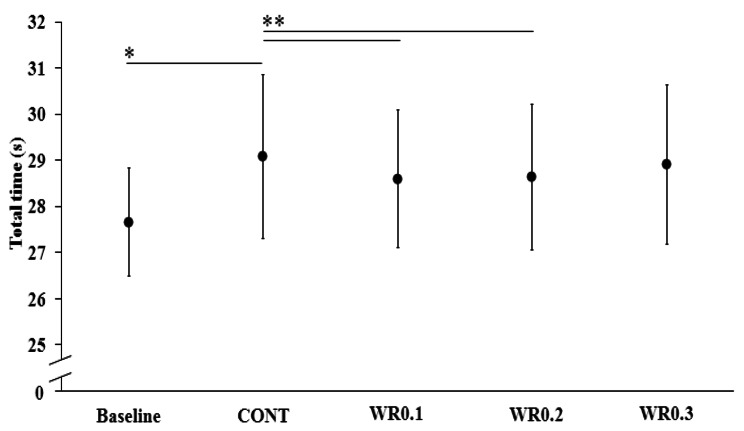
RCoD performances of WR0.1, WR0.2, WR0.3 and unloaded (CONT) warm-up conditions at 4 time points: during pre-warm-up intervention measurements (baseline) and at SSG1-8 min, SSG1-15 min (first HT) and SSG2-15 min (second HT) post-warm-up intervention. *p < 0.05, **p < 0.01

ANOVA revealed significant differences between the three time points (SSG1-8 min, SSG1-15 min and SSG2-15 min). However, the post-hoc tests displayed that there was a less pronounced rate of match-play induced decrease in repeated CoD performance (for both and total times) with WR0.1 and WR0.2 comparatively to WR0.3 and CONT conditions (*p* < 0.05). (Tables 3 and 4). A significant decrease in repeated CoD performance was observed during SSG1-8 min (*p* < 0.05; small ES: 0.35), SSG1-15 min (*p* < 0.01; medium ES: 0.7), and SSG2-15 min (*p* < 0.01; large ES: 0.85) for both loaded and unloaded conditions (Table 2) compared to baseline. For both loaded and unloaded conditions, better repeated CoD’s total time values were collected during SSG1-8 min (*p* < 0.05) compared to SSG1-15 min and SSG2-15 min, with better repeated CoD performance values in SSG1- 15 min, compared to SSG2-15 min (Fig. 2). WR0.1, and WR0.2 significantly affected the pattern of repeated CoD’s mean peak time decrement during SSG1 and SSG2 through dampening the observed rate of decrease in the unloaded condition CONT compared to baseline (Tables 3 and 4).

**Table 3:** Descriptive statistics (mean ± SD) and comparison of individual performances in the peak time for repeated change of direction performance (RCoD) following loaded interventions (WR0.1, WR0.2, WR0.3), and in unloaded warm-up conditions (CONT)

Condition	CONT	WR0.1	WR0.2	WR0.3
Baseline	6.95 ± 0.29	6.89 ± 0.29	6.88 ± 0.29	6.91 ± 0.30
SSG1-8 min	7.22 ± 0.51	7.05 ± 0.45^**a,b**^	7.11 ± 0.46^**a**^	7.17 ± 0.42^**c**^
SSG1-15 min	7.40 ± 0.48	7.26 ± 0.38^**a**^	7.27 ± 0.42^**a**^	7.31 ± 0.43 ^**a**^
SSG2-15 min	7.52 ± 0.50	7.38 ± 0.33^**b**^	7.39 ± 0.43	7.47 ± 0.44^**c**^

**Note**: ^a^significantly different from CONT (*P* < 0.01), ^s^ignificantly different from LWBR0.3 (*P* < 0.01), ^c^significantly different from LWBR0.1 (*P* < 0.05)

**Table 4:** Descriptive statistics (statistical significance, magnitude and effect sizes: ES) for the mean repeated change of direction performance (repeated CoD) following loaded interventions (WR0.1, WR0.2, WR0.3), and under unloaded conditions (CONT)

	SSG1-8 min	SSG1-15 min	SSG2-15 min
CONT vs. RW0.1 P ES (Cohen’s d)	0.0014 0.35 (small)	0.0027 0.79 (medium)	0.0100 0.28 (small)
CONT vs. RW0.2 P ES (Cohen’s d)	0.0196 0.24 (small)	0.0018 0.28 (small)	0.0033 0.28 (small)
CONT vs. RW0.3 P ES (Cohen’s d)	0.4203 0.07 (trivial)	0.0464 0,19 (trivial)	0.0955 0.11(trivial)
RW0.1vs RW0.2 P ES (Cohen’s d)	0.0023 0.78 (medium)	0.7794 0.06 (trivial)	0.7072 0.08 (trivial)
RW0.1vs RW0.3 P ES (Cohen’s d)	0.0007 0.30 (small)	0.1347 0.12 (trivial)	0.0195 0.17 (trivial)

WR: Wearable Resistance condition; CONT: control condition

On the other hand, there were no significant differences in repeated CoD times between CONT and WR0.3. But, a small effect on repeated CoD performance was observed in WR0.2 (*p* < 0.05; small ES: 0.28) during SSG2-15 min compared to CONT (Tables 3 and 4).

**Fig. 3:**
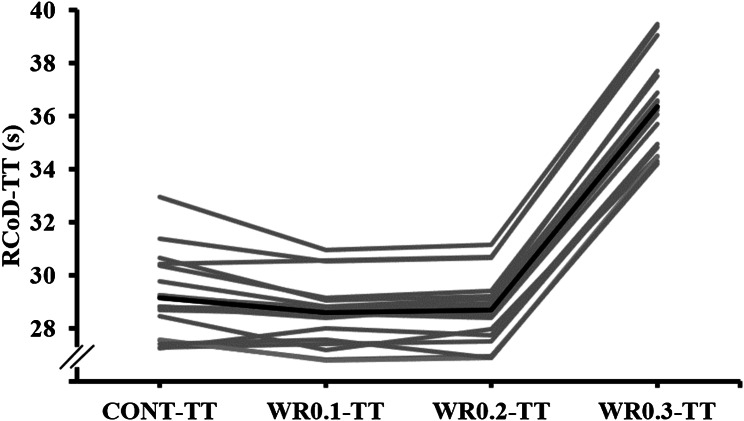
Comparison of individual performances in the peak time of RCoD following the loaded and the unloaded warm-up conditions

## Discussion

This study explored the extent to which different preconditioning lower limb WR micro-loadings influenced fluctuations in repeated CoD performance while playing SSG in young post-pubertal male players. The main findings showed that WR0.1, and WR0.2 conditions positively affected the pattern of repeated CoD performance mean, and peak time decreases throughout the first (SSG1), and the second (SSG2) halves of the play. These conditions also dampened the decrease of repeated CoD performance, suggesting their superiority compared to WR0.3 and CONT. Previous studies on WR warm-ups with lower limb loading demonstrated significant acute effects on jumping, running and sprinting with loads of between 0.3 and 8.5% body mass [38].

Our findings are in line with previous investigations showing beneficial effects of lower body WR warm-ups on sprint acceleration performance in nineteen male rugby athletes [39], punching power, jumping height, bench press and 5RM half-squats in seventeen amateur male combat athletes [40, 41]. In a more recently published protocol, thirty-one young national level soccer players wore lower body WR with loading of 200 to 600 g during the warm-up [15]. The results indicated that WR training improved pre-training to post-training 10 and 20 m sprint times more than in the unloaded warm-up program [15], which is partially in accordance with our findings. A recent systematic review of longitudinal studies assessing the effects of lower limb WR on sprint running performance concluded that lower limb WR overloads sprint movement velocity that could stimulate increases in horizontal force output, indicated that lower limb WR has the potential to improve sprinting performance [17].

Recent reports confirm the benefits of WR on the lower limbs WR during warm-ups in mixed martial arts [38], and in elite soccer players [15]. Nevertheless, and to the best of our knowledge, this study is the first to explore the sustained effects of lower limb WR preconditioning protocols on repeated CoD performance during a soccer game by young players. Such findings could have important implications for strength and conditioning practices given the important similarities that exist between a competitive game and SSG.

When dealing with lower limb conditioning strategies in young participants, the choice placement of the load as well as its magnitude should be made with caution [21]. Changes in biomechanical and limb inertia are more noticeable when load increases [21], and when the load is located in a more distal position on the limb (17; 42). Research on the longitudinal effects of pregame warm-ups using WR in the lower limbs of soccer players of different ages and levels of expertise.

A number of physiological factors could explain the dampening of the fatigue-induced natural process of decreases in repeated CoD performance during play (up to the 40th minute post warm-up) after WR0.1 and WR0.2. We speculate that these factors are likely linked to acute increases in leg stiffness [42] caused by force contribution of the stretch-shortening cycle [43]. According to Feser et al. [22], resistance sprint training goals should increase lower limb strength and neural activation while avoiding adverse effects of sprint training. Given that WR specific movement training is an example of the application of training specificity and transference [39], an enhanced coordination of movement may have contributed to these outcomes. The WR loads should induce acute beneficial effects such that coordination can be adjusted in response to movement variability. Given the fact that the participants in our study were engaged in SSGs, they were playing a modified version of the game that nevertheless maintained the fundamental technical dynamics and specificities of their sport [6]. It is unknown if the mechanisms cited above are applicable to competitive games. Future research should explore the mechanisms by which conditioning WR dampens decreases in performances throughout the two halves of a soccer match.

The results of study indicated a substantial variability in individual responses (Fig. 3), suggesting that the practical utility of WR for dampening the repeated CoD decreases during a game is likely to also be individual-dependent. Coaches should appreciate the range of individual responses and use our findings to tailor individual WR prescriptions in to players on the field. Future research should also explore the optimal lower limb WR placement, orientation, and magnitude or WR load (or increase exposure times to the load) to further investigate the potential performance benefits of this promising warm-up modality. Additionally, further research is needed to investigate performance adaptations that may occur following an extended period (long-term effect) of using WR.

One limitation of our study is that repeated CoD performance was assessed during SSG that do not accurately replicate conditions of a competitive soccer match. Future research should explore optimal loading and placement of lower limb WR to the assess the ability of WR to dampen loss of athletic performances in subsequent soccer games. Another area of interest is to investigate the benefits of WR on a wider range of soccer performance markers.

## Conclusions

Our study reports that strength and conditioning trainers should consider including micro-loadings WR corresponding to 0.1 or 0.2% of players’ body mass during the later phases of warm-up practices. This pre-competition preconditioning activity could dampen the fatigue-induced decreases in repeated CoD performance during a competitive soccer game.

## Data Availability

The datasets generated during and analyzed during the current study are not publicly available due to confidential information about the participants but are available from the corresponding author on reasonable request.
